# Editorial: Functional units of nanocrystals: Synthesis, tailoring, and applications

**DOI:** 10.3389/fchem.2022.1087379

**Published:** 2022-11-18

**Authors:** Jiahui Lin, Huixian Wei, Jingtao Su, Xinyuan Li, Jintao Huang, Shoaib Anwer, Shuyu Liang, Guangtao Cong, Linli Xu, Fushen Lu, Muwei Ji

**Affiliations:** ^1^ Department of Polymeric Materials and Engineering, School of Materials and Energy, Guangdong University of Technology, Guangzhou, China; ^2^ College of Chemistry and Chemical Engineering, Chemistry Department, Shantou University, Shantou, China; ^3^ MOE Key Laboratory of Cluster Science, School of Chemistry and Chemical Engineering, Beijing Institute of Technology, Beijing, China; ^4^ Department of Mechanical Engineering, College of Engineering, Khalifa University, Abu Dhabi, United Arab Emirates; ^5^ Sika Aktien Aktien Gesellschaft (AG), Baar, Switzerland; ^6^ Institute of Low-Dimensional Materials Genome Initiative, College of Chemistry and Environmental Engineering of Shenzhen University, Shenzhen, China; ^7^ Department of Applied Biology and Chemical Technology, The Hong Kong Polytechnic University Shenzhen Research Institute, The Hong Kong Polytechnic University, Hong Kong SAR, China

**Keywords:** functional units, nanocrystals, synthesis, tailoring, applications

Functional materials with special structural units are explored to acquire excellent properties; highly ordered functional units of atomic or molecular sizes would provide a high effective atom utilization and a superior functional synergetic effect. For functional materials, their active sites, or main functional structures, can be defined by defects, high-index facets, single-atom catalytic sites with coordinated surroundings, doping sites, heterostructure interfaces, frameworks, etc. Herein, these sites or structures are further defined as functional units with sizes ranging from more than 10 p.m. (such size may be one atom) to sub-micrometer, often determining their capacities for catalysis, energy conversion and storage, and luminescence (Lu and Yin, 2012; Gaitzsch et al., 2016; Cao et al., 2022). We hope that “functional units” are helpful to more clearly explain structure-property relationships, providing additional possibilities for designing and developing new materials with novel functionalities. For example, single-atom sites for catalysis and new frameworks for harvesting solar energy are considered functional units. Nowadays, various functional units have been proposed in different fields for exploring materials with significantly enhanced functional properties; however, it is difficult to characterize confined structures and to reveal functional relationships between structures.

Li and coauthors reviewed the atomic surface modulation of 2D semiconductors for enhancing photocatalysis; discussing vacancies, single atom-doping, and dual-site components. For 2D semiconductor photocatalysts, there are several effective paths to promote their performance. Li et al. first focused on vacancy modulation in various 2D semiconductors to tune the band gap and to enhance charge transfer during photocatalysis. Either modulating the band gap or enhancing charge transfer reduced the number of photo-generated electrons and holes, which play a key role in photocatalysis. Photocatalysis was also enhanced by introducing single-atom sites that shorten the migration distance of photo-generated charges. More importantly, these introduced single atoms are widely accepted as efficient photocatalytic sites. Meanwhile, catalytic sites in 2D semiconductors were also modulated to form dual sites: metal-metal or metal-vacancies sites. The metal-metal dual site on the surface of 2D semiconductors allowed modulation of the d band center and its position relative to the Fermi level, which affected the absorption energy of the reactive metal and facilitates electron transfer. Metal single-atom loading also resulted in metal-vacancy dual sites for catalysis, and the coupling effect between the metal and the vacancy promoted photocatalysis selectivity. Separating the photo-generated electrons and holes is crucial in photocatalysis and constructing such functional units for enhancing the photocatalysis remains a major challenge.

Iqbal et al. reported ZnFe_2_O_4_/S-g-C_3_N_4_ with high photocatalytic activity obtained by boosting the separation of photo-generated electrons and holes. The proposed heterostructure facilitated photo-generated charge transfer and thus enhanced photo-degradation and antimicrobial performances. This work on constructing heterostructures for promoting photocatalysis also illustrates the importance of interface structure in accelerating electron and hole transfer.

Xing and coworkers proposed different scenarios for catalysis, based on the interaction interface between a metal and carbon. The authors described four types of metal-carbon interfaces and their capacities for catalysis. Regarding the metal-carbon interaction surface, generated using large metal nanoparticles, the carbon support played a role in preserving the aggregation of metal NPs and the catalytic activity depended on the metal components. In the case of single-atom catalysts, the carbon support provided a coordination microenvironment for the metal atom, and limited the catalytic activity. For metal@carbon core-shell catalysts, electrons in the metal were transferred to carbon, and the structure allowed maintenance of the high activity of metal nanoparticles, thereby preventing corrosion in harsh environments. Meanwhile, the metal-carbon interface formed a Mott-Schottky junction that also modulated electron transfer and therefore the associated catalytic activities. These works clearly presented well-defined interfaces for promoting catalysis.

Zhang et al. prepared Pt-based nanosheets to enhance the hydrogenation catalysis of olefins and nitrobenzene. The high performances originated from the high surface area of the Pt nanosheets as well as the interconnected network, which provided a high concentration of active sites. The TOFs of the as-prepared Pt nanosheets were three times higher than those of homemade Pt nanoparticles. The morphologies and the interconnected sites were considered key structures for hydrogenation catalysis. Although the authors did not indicate the functional units for catalysis, hydrogenation sites were conceived to be dependent on the unique structure.

This Research Topic for Frontiers in Chemistry aims to navigate structural units for promoting catalytic activities. Besides catalysts, functional units are also expected to be applied in other functional materials. Last but not least, we believe that this topic will educate readers and inspire scientists working in the fields of chemistry and materials engineering to identify and investigate structural units that influence the associated functions.
